# Anti-hypertensive medications and erectile dysfunction: focus on β-blockers

**DOI:** 10.1007/s12020-024-04020-x

**Published:** 2024-09-13

**Authors:** G. Corona, W. Vena, A. Pizzocaro, G. Salvio, C. Sparano, A. Sforza, M. Maggi

**Affiliations:** 1 Endocrinology Unit, Maggiore Hospital, Azienda-Usl Bologna, Bologna, Italy; 2 Department of Biomedical Sciences, Humanitas University, Rozzano, Milan, Italy; 3 Diabetes Center, Humanitas Gavezzani Institute, Bergami, Italy; 4 Unit of Endocrinology, Diabetology and Medical Andrology, IRCSS, Humanitas Research Hospital, Rozzano, Milan, Italy; 5 Endocrinology Clinic, Department of Clinical and Molecular Sciences, Polytechnic University of Marche, Ancona, Italy; 6 Endocrinology Unit Department of Experimental, Clinical and Biomedical Sciences, University of Florence, Florence, Italy

**Keywords:** Erectile dysfunction, Hypertension, Antihypertensive medications, Beta-blockers

## Abstract

**Purpose:**

Although anti-hypertensive medications, including thiazides and β-blockers (BBs) in particular, have been suggested to cause erectile dysfunction (ED) their real contribution is still conflicting. The aim of this paper is to summarize available evidence providing an evidence-based critical analysis of the topic.

**Methods:**

An overall comprehensive narrative review was performed using Medline, Embase and Cochrane search. In addition, to better understand the impact of BBs on ED a specific systematic review was also performed.

**Results:**

The negative role of centrally acting drugs, such as clonidine and α-methyldopa, is well documented althuogh limited controlled trials are available. Angiotensin-converting enzyme inhibitors (ACEis), angiotensin receptor blockers (ARBs), and calcium-channel-blockers (CCBs) have neutral (CCBs) or even positive (ACEis and ARBs) effects on erectile function. Despite some preliminary negative reports, more recent evidence does not confirm the negative impact of thiazides. BBs should be still considered the class of medications more often associated with ED, although better outcomes can be drawn with nebivolol.

**Conclusion:**

Sexual function should be assessed in all patients with arterial hypertension, either at diagnosis or after the prescription of specific medications. A close related patient-physician interaction and discussion can overcome possible negative outcomes allowing a successful management of possible side effects.

## Introduction

Arterial hypertension (AH) represents a well-recognized public health issue estimated to affect over one billion individuals worldwide [1] as well as an established risk factor for the development of cardiovascular disease (CVD), chronic kidney disease (CKD), cognitive decline and premature mortality [2, 3]. The blood pressure (BP) lowering effect of antihypertensive medications offers considerable benefits in reducing the impact of such morbidities and related mortality [4]. However, evidence about their possible negative impact on male sexual function is still the object of an intense debate [5, 6]. The Treatment of Mild Hypertension Study (TOMHS) was one of the first large population trials describing an association between AH and sexual dysfunction in both male and female participants [7]. Since then, a large body of evidence clarified how the presence of AH is associated to an increased risk of erectile dysfunction (ED) across different study populations [8–12]. Accordingly, available data suggest that ED is two-times higher in subjects with AH when compared to those derived from the general population [13]. Several pathogenetic mechanisms have been suggested including either central (e.g. catecholamine depletion) or peripheral mechanisms (e.g. metabolic and hormonal profile impairment; see also below) [5, 6, 14]. Notably, antihypertensive medications such as thiazide and β-blockers (BBs) have been found, more frequently than others, to play a detrimental role on sexual function [2, 6, 13, 14]. Calcium-channel-blockers (CCBs) or α-blockers (ABs) have shown essentially neutral effects on ED [6, 14], although negative role of α-blockers on ejaculatory function have been reported [15]. Conversely, angiotensin receptor blockers (ARBs) have been suggested to exert the most favourable impact on erectile function [2, 6, 14]. However, it should be recognized that the available evidence is still poor and often derived from expert opinion, rather than evidence-based data. Similarly, the net impact of BBs on male sexual function in men suffering AH is still a matter of debate. While some older trials, based on self-reported erectile function, pointed towards a possible sexual impairment in both healthy and hypertensive men [16, 17], others, based on standardized erectile function assessment methods (International Index of Erectile Function or IIEF), could not confirm such an association [18]. Similarly, a large network meta-analysis including 25 studies accounting for more than 7700 patients showed a neutral effect on erectile function [5]. In addition, there is evidence suggesting that patient knowledge and prejudice about BB side effects could represent a major determinant of ED onset [19]. Moreover, the different pharmacodynamic profiles of BB action are likely responsible for the different impacts on sexual function observed when different molecules are compared; indeed working evidence showed how nebivolol, a third generation BB, is rather associated with slight improvement [20] or no impact on patient IIEF (see below [21]).

The aim of the present review is to provide a summary of the available data related to the possible association between ED and the use of AH medications with a particular focus on the impact of BBs.

## Methods

A comprehensive narrative review was performed using Medline, Embase and Cochrane searches and including the following words: ((“erectile dysfunction” [MeSH Terms] OR (“erectile” [All Fields] AND “dysfunction” [All Fields]) OR “erectile dysfunction” [All Fields]) AND (“hypertense” [All Fields] OR “hypertension” [MeSH Terms] OR “hypertension” [All Fields] OR “hypertension s” [All Fields] OR “hypertensions” [All Fields] OR “hypertensive” [All Fields] OR “hypertensive s” [All Fields] OR “hypertensives” [All Fields]) AND (“medic” [All Fields] OR “medical” [All Fields] OR “medicalization” [MeSH Terms] OR “medicalization” [All Fields] OR “medicalizations” [All Fields] OR “medicalize” [All Fields] OR “medicalized” [All Fields] OR “medicalizes” [All Fields] OR “medicalizing” [All Fields] OR “medically” [All Fields] OR “medicals” [All Fields] OR “medicated” [All Fields] OR “medication s” [All Fields] OR “medics” [All Fields] OR “pharmaceutical preparations” [MeSH Terms] OR (“pharmaceutical” [All Fields] AND “preparations” [All Fields]) OR “pharmaceutical preparations” [All Fields] OR “medication” [All Fields] OR “medications” [All Fields])) AND ((humans[Filter]) AND (male[Filter]) AND (english[Filter])). Publications from January 1st, 1969 up to February 29th, 2024 were included.

In addition, to better analyze the impact of BBs on ED a specific systematic review was performed using the same time period reported above, and the following words ((“erectile dysfunction” [MeSH Terms] OR (“erectile” [All Fields] AND “dysfunction” [All Fields]) OR “erectile dysfunction” [All Fields]) AND (“adrenergic beta antagonists” [Pharmacological Action] OR “adrenergic beta antagonists” [MeSH Terms] OR (“adrenergic” [All Fields] AND “beta antagonists” [All Fields]) OR “adrenergic beta antagonists” [All Fields] OR (“beta” [All Fields] AND “blockers” [All Fields]) OR “beta blockers” [All Fields])) AND ((humans [Filter]) AND (male [Filter]) AND (English [Filter])) (Appendix 1). In particular, a meta-analytic approach was selected in order to minimize possible sources of bias derived from a personal interpretation of the data. Meta-analysis was performed using Comprehensive Meta-analysis Version 2, Biostat, and (Englewood, NJ, USA). Multivariate analyses as well as other analyses were performed on SPSS (Statistical Package for the Social Sciences; Chicago, USA) for Windows, version 25.

Clinical data were derived from a consecutive series of more than 4000 patients seeking medical care at the University of Florence, as previously described [3, 11, 22, 23].

### Antihypertensive drugs and ED in symptomatic patients

ED subjects represent a population enriched with associated morbidities such as metabolic and hormonal derangements, as well as traditional CV risk factors, including AH [12, 24–26]. Accordingly, ED is nowadays considered an early marker of forthcoming major CV diseases (MACE) either in the general population [27] or in patients with diabetes mellitus [28]. In a large series of patients seeking medical care for ED, we previously demonstrated that subjects in the lowest pulse pressure quartile (20–45 mmHg) had up to a 60% reduced risk of MACE after a mean follow-up of 4.3 years [3]. In order to better understand the impact of the most important AH medications, we here reported a specific analysis in an updated population, including 3903 subjects with a mean age of 50.6 ± 11.5 years (Table 1 and 3). Only the first-line drugs recommended by the European Society of Hypertension (ESH) were investigated [2]. The subjects were evaluated according to what was previously described [3, 11]. Among them, 25.2% had a history of AH or were taking AH medications. In particular, the use of BBs, angiotensin-converting enzyme inhibitors (ACEis), ARBs, CCBs, diuretics and ABs was reported by 8.5%, 12.8%, 9.3%, 9%, 10.9%, and 6.5%, respectively. Specific correlates of any of the aforementioned classes of anti-hypertensive medications were assessed by binary logistic regression analysis, using as a reference those not taking any medication and after adjustment for confounders (including age, smoking and alcohol consumption, history of AH and CVD and body mass index, BMI). As shown in Table 1, no difference in the reported rate of erection sufficient for penetration was observed for all the classes investigated. In BBs, but no in other classes, use was inversely related to the reported frequency of intercourse. In addition, BBs along with ACEis, CBBs and ABs showed the best impact in improving flaccid acceleration at Penile Colour Doppler Ultrasound (PCDU). Similarly, BBs and ARBs showed the best impact in reducing pulse pressure, which is considered one of the best clinical markers of endothelial function [3, 11]. Several AH medications, including BBs, ARBs and CBBs, were associated with a reduction in testis volume. This finding was tightly related to the observed reduction in T circulating levels in patients taking BB and ARBs. Conversely, no modification in gonadotropins levels was observed, apart from a slight, although statistically significant, increase in FSH observed with the use of CBBs. Finally, the use of several classes of AH medications was more often associated with an increase in anxious and depressive symptomatology, as assessed by the Middlesex Hospital Questionnaire, a tool to investigate psychological disturbances in a non-psychiatric setting [29] (Table 1).

**Table 1:** Impact of several arterial hypertension medications on several clinical and instrumental parameters

Item	Type of drug	OR [95% CI]	*p*
**Sexual function parameters**			
Erection sufficient for intercourse	*β-blockers*	0.829 [0.557; 1.235]	0.357
	*AT II receptor blockers*	0.748 [0.505; 1.109]	0.149
	*Calcium channel blockers*	1.045 [0.716; 1.524]	0.821
	*ACEis*	0.844 [0.584; 1.220]	0.366
	*Diuretics*	0.877 [0.584; 1.317]	0.526
	*α-blockers*	0.935 [0.798; 1.096]	0.408
Frequency of intercourse	***β-blockers***	**0.818 [0.695; 0.962]**	**0.015**
	*AT II receptor blockers*	1.001 [0.845; 1.187]	0.987
	*Calcium channel blockers*	0.940 [0.799; 1.105]	0.451
	*ACEis*	0.944 [0.806; 1.105]	0.473
	*Diuretics*	0.911 [0.771; 1.077]	0.275
	*α-blockers*	1.036 [0.855; 1.255]	0.717
Flaccid PSV (cm/sec) at PCDU	*β-blockers*	1.027 [0.994; 1.061]	0.107
	*AT II receptor blockers*	1.025 [0.990; 1.061]	0.157
	*Calcium channel blockers*	1.018 [0.984; 1.053]	0.313
	*ACEis*	1.012 [0.980; 1.044]	0.477
	*Diuretics*	1.002 [0.969; 1.036]	0.909
	***α-blockers***	**1.044 [1.006; 1.083]**	**0.021**
Flaccid acceleration (m/s^2^) at PCDU	***β-blockers***	**1.163 [1.001; 1.352]**	**0.048**
	*AT II receptor blockers*	1.030 [0.880; 1.207]	0.710
	***Calcium channel blockers***	**1.183 [1.019; 1.375]**	**0.028**
	***ACEis***	**1.362 [1.176; 1.577]**	**0.0001**
	***Diuretics***	**1.175 [1.004; 1.376]**	**0.044**
	***α-blockers***	**1.551 [1.304; 1.844]**	**0.000**
Dynamic PSV (cm/sec) at PCDU	*β-blockers*	1.003 [0.993; 1.013]	0.602
	*AT II receptor blockers*	0.994 [0.984; 1.005]	0.305
	*Calcium channel blockers*	1.001 [0.991; 1.011]	0.844
	*ACEis*	1.008 [0.999; 1.018]	0.081
	*Diuretics*	1.006 [0.996; 1.017]	0.230
	***α-blockers***	**1.020 [1.009; 1.032]**	**0.001**
**Clinical parameters**			
Testis volume (ml)	***β-blockers***	**0.952 [0.922; 0.984]**	**0.003**
	***AT II receptor blockers***	**0.948 [0.916; 0.980]**	**0.002**
	***Calcium channel blockers***	**0.964 [0.933; 0.996]**	**0.028**
	*ACEis*	0.986 [0.955; 1.019]	0.337
	*Diuretics*	0.981 [0.948; 1.015]	0.273
	*α-blockers*	0.991 [0.954; 1.031]	0.665
Pulse Pressure (mmHg)	***β-blockers***	**0.985 [0.973; 0.9963]**	**0.009**
	***AT II receptor blockers***	**0.988 [0.977; 0.999]**	**0.039**
	*Calcium channel blockers*	1.006 [0.995; 1.017]	0.278
	*ACEis*	0.991 [0.980; 1.003]	0.141
	*Diuretics*	0.995 [0.984; 1.007]	0.404
	*α-blockers*	0.995 [0.982; 1.008]	0.439
**Hormonal parameters**			
Total testosterone (nmol/L)	***β-blockers***	**0.965 [0.939; 0.993]**	**0.013**
	***AT II receptor blockers***	**0.961 [0.933; 0.989]**	**0.007**
	*Calcium channel blockers*	0.975 [0.949; 1.001]	0.059
	*ACEis*	1.011 [0.985; 1.037]	0.413
	*Diuretics*	0.974 [0.946; 1.002]	0.073
	*α-blockers*	0.972 [0.939; 1.005]	0.099
FSH (mU/L)	*β-blockers*	1.292 [0.806; 2.072]	0.288
	*AT II receptor blockers*	0.898 [0.551; 1.462]	0.664
	***Calcium channel blockers***	**1.929 [1.192; 3.121]**	**0.007**
	*ACEis*	1.480 [0.918; 2.387]	0.108
	*Diuretics*	0.872 [0.530; 1.434]	0.589
	*α-blockers*	1.318 [0.750; 2.317]	0.338
LH (mU/L))	*β-blockers*	0.875 [0.528; 1.448]	0.602
	*AT II receptor blockers*	0.868 [0.514; 1.466]	0.596
	*Calcium channel blockers*	1.176 [0.703; 1.968]	0.536
	*ACEis*	1.182 [0.755; 2.177]	0.358
	*Diuretics*	0.901 [0.528; 1.537]	0.707
	*α-blockers*	1.041 [0.552; 1.965]	0.900
**Psychological (MHQ) parameters**			
Anxiety symptoms (MHQ-A)	***β-blockers***	**1.079 [1.032; 1.127]**	**0.001**
	***AT II receptor blockers***	**1.094 [1.044; 1.147]**	**0.0001**
	*Calcium channel blockers*	1.028 [0.983; 1.075]	0.228
	*ACEis*	1.009 [0.967; 1.054]	0.673
	***Diuretics***	**1.072 [1.023; 1.123]**	**0.003**
	***α-blockers***	**1.077 [1.023; 1.133]**	**0.005**
Phobic symptoms (MHQ-P)	***β-blockers***	**1.136 [1.074; 1.201]**	**0.0001**
	***AT II receptor blockers***	**1.127 [1.062; 1.195]**	**0.0001**
	***Calcium channel blockers***	**1.064 [1.006; 1.126]**	**0.031**
	*ACEis*	1.053 [0.996; 1.112]	0.067
	***Diuretics***	**1.105 [1.043; 1.170]**	**0.001**
	***α-blockers***	**1.151 [1.080; 1.227]**	**0.000**
Somatic symptoms (MHQ-S)	***β-blockers***	**1.079 [1.021; 1.139]**	**0.006**
	***AT II receptor blockers***	**1.113 [1.050; 1.179]**	**0.0001**
	*Calcium channel blockers*	1.056 [0.999; 1.116]	0.056
	*ACEis*	1.026 [0.972; 1.083]	0.353
	***Diuretics***	**1.103 [1.043; 1.168]**	**0.001**
	***α-blockers***	**1.148 [1.082; 1.218]**	**0.000**
Depressive symptoms (MHQ-D)	***β-blockers***	**1.096 [1.044; 1.151]**	**0.0001**
	***AT II receptor blockers***	**1.125 [1.068; 1.185]**	**0.0001**
	***Calcium channel blockers***	**1.079 [1.027; 1.134]**	**0.003**
	*ACEis*	1.032 [0.985; 1.082]	0.188
	***Diuretics***	**1.097 [1.043; 1.155]**	**0.0001**
	***α-blockers***	**1.123 [1.063; 1.187]**	**0.000**

Data were derived from 3903 subjects seeking medical care at our unit for erectile dysfunction as previously reported [3, 11]

*ACEi* angiotensin-converting enzyme inhibitors, *PSV* peak systolic velocity, *PDU* penile doppler ultrasound, *ATII* Angiotensin II receptor, *MHQ* Middlesex Hospital Questionnaire

Bold values: statistically significance

### Antihypertensive drugs and ED in the general population

As reported in the introduction, the specific impact of anti-hypertensive medications on ED is still confusing and based on poor-quality data. A summary, performed more than 10 years ago, based on 12 different hypertension clinical practice guidelines, showed that only a few of them (*n* = 3) recognised the importance of assessing sexual function prior to initiation and/or follow-up of antihypertensive therapy, and only two provided specific management recommendations [30]. The European Society of Hypertension (ESH) has recognized the importance of ED since 2007 by introducing a specific Working Group on Sexual Dysfunction [31]. In its updated guideline [2] and in a position paper [13], ESH still supports the negative role of BBs and diuretics and the better outcomes of ARBs and nebivolol on ED. In the following sections, a summary of the available data related to the most important classes of anti-hypertensive medications will be provided (see also Table 2).

**Table 2:** Summary of the effects of anti-hypertensive medication on erectile function

Antihypertensive medication	Effect on eretile function	Quality of evidence
Centrally acting drugs		
Clonidine	*↓↓*	+
Methyldopa	*↓↓*	+
Diuretics		
Thiazides	***-/****↓*	**+++**
Loop	***-***	**+**
Potessium-sparing	*↓↓*	**++**
Calcium-Channel blockers		
Amplodipine	-	**++**
Nifedipine	-	**+**
Verapamil	*-*	+
α-blockers	***-***	***+***
ACE-inhibitors/angiotensin-receptor blockers		
ACE-inhibitors	-/↑	**+++**
Angiotensin-receptor blockers	-/↑	**+++**
Β-blockers		
Non-selective	*↓*	**++**
Selective	***-/****↓*	++
Nebivolol	-/↑	+++

*ACE* angiotensin-converting enzyme; ↑ = positive effect; ↓ = negative effect; - = neutral effect. A higher number of + indicates better quality of the evidence

#### Central acting-drugs: *clonidine and α-methyldopa*

*Clonidine* acts by stimulating α-adrenergic receptors resulting in inhibition of bulbar sympathetic vasoconstrictor and cardio-accelerator centres. Similarly, *α-metildopa* can negatively interfere with the sympathetic nervous system inducing catecholamine depletion at a central level. The impairment of sympathetic tone at a central level represents the main putative negative effect of these drugs on ED [32]. Accordingly, the prevalence of ED has been reported in up to 70% of subjects treated with clonidine [33] and up to 53% of those taking methyldopa [34]. In line with these data, evidence derived from animal models showed that clonidine impairs sexual behaviour and the nerve-mediated response to erection in rats [35]. However, it should be recognised that data related to these drugs are derived from very old and uncontrolled reports, often using only self-reported data and not specific validated questionnaires for the evaluation of ED.

#### Diuretics

Several uncontrolled reports support the negative role of thiazides on erectile function [36, 37]. The working mechanism of action is based on the possible stimulation of the central α-adrenergic receptor pathway due to sodium depletion [38]. In line with this hypothesis, a prospective five-year study performed in the general population showed that the use of thiazides was associated with 2.4 fold increased risk of ED, even after the adjustment for confounders [36]. In contrast to this study, data derived from the Massachusetts Male Aging Study (MMAS) did not confirm an association between ED and the use of thiazides in the multivariate analysis [39]. A first randomized controlled trial (RCT) showed that chlorthalidone impaired erectile function when compared to placebo after 6 months [40]. However, these data were not confirmed in a two-year larger TOMHS study including more than 900 subjects [7]. The contribution of thiazides in combination with other treatments on ED is difficult to assess. However, a recent network meta-analysis, including nine studies with at least one thiazide harm, did not provide an increased ED-related risk [5]. The use of aldosterone antagonists, such as spironolactone and eplerenone, potentially negatively interferes with sexual function due to their anti-androgenic effects and their possible negative role in gonadotropins secretion [38]. However, specific placebo RCTs are still lacking. Similarly, information related to the effects of loop-diuretics on ED is limited. Interestingly, data derived from the MMAS confirmed an increased risk of ED related to the use of either loop-diuretics or spironolactone, even after adjustment for confounders [39].

#### Calcium-channel blockers

Some preliminary studies indicated that nifedipine was associated with worse erectile function when compared to placebo in patients with hypertension [41] or coronary artery diseases [42]. The latter observation, however, was not confirmed when multiple drug treatments where investigated. Accordingly, the TOMHS trial did not find any difference when acebutolol, amlodipine, chlorthalidone, doxasosin, or enalapril were compared to placebo [7]. In addition, “in vitro” studies indicated that nifedipine, diltiazem, and verapamil were able to counteract norepinephrine-induced contraction of corona cavernosa smooth muscles cells [43]. Similar data were derived from amlodipine in hypertensive rats [44]. However, no sufficient data in humans are available to support the positive effect of calcium-channel blockers (CCB) on erectile function. Accordingly, the available network meta-analysis, including five RCTs with at least one CCBs arm, found a neutral effect of these medications on ED rate [5].

#### Alpha-blockers

According to the current guidelines ABs represent the 3rd line therapy in the overall population [2]. Data derived from the TOMHS trial showed no difference in ED rate when compared to placebo either after 24 or 28 months. However, a trend towards a lower incidence of ED in patients treated with ABs was detected [7]. The combination between ABs with phosphodiesterase type 5 inhibitors (PDE5i) did not improve ED in the overall general population [45]. Conversely, the combined therapy resulted in better IIEF score when patients with low urinary tract symptoms were considered [46].

#### ACE-inhibitors and angiotensin-receptor blockers

The TOMHS trial [7] did not find any advantage of enalapril, when compared to placebo, on erectile function. Conversely, a more recently published trial, performed in 59 subjects with atherosclerotic ED, showed that quinapril was associated with an improvement of penile vascular flow and ED, as assessed by IIEF, when compared to placebo [47]. Similarly, several uncontrolled and open-label reports have suggested an improvement of erectile function related to the use of ARBs (see for review [6, 13, 14, 38]). Several mechanisms related to the renin-angiotensin pathway can support the positive role of either ACEìs or ARBs on erectile function. Angiotensin II is produced and released by endothelial and smooth muscle cells of the penile arteries and corpora cavernosa under ACE control. At this level, angiotensin II is involved in the regulation of penile detumescence through a direct action on endothelial cells and indirectly by the stimulation of phosphodiesterase type 5 (PDE5) [48]. Furthermore, angiotensin II is involved in the increase of the production of reactive oxygen species through the stimulation of the AT1-receptor and then nicotinamide adenine dinucleotide phosphate (NADPH) oxidase [49]. Despite this evidence, only a few RCTs on the impact of ARBs are available. A recent meta-analysis including four placebo-controlled trials and 2809 subjects concluded that the use of ARBs was associated with an overall improvement in sexual activity, but erectile function did not increase significantly [50]. Similarly, a network meta-analysis, including five studies with at least one ACEi harm and eight studies with at least one ARB harm, concluded with a neutral effect of both medication classes when compared to placebo [5].

#### Beta-blockers

The use of BBs has been frequently associated with worse erectile function. However, their real contribution is still conflicting and mainly derived from uncontrolled observations. In order to better clarify their role on ED and male sexual function, we systematically investigated date derived from epidemiological studies as well as those obtained in RCTs.

##### Epidemiological data

**Fig. 1:**
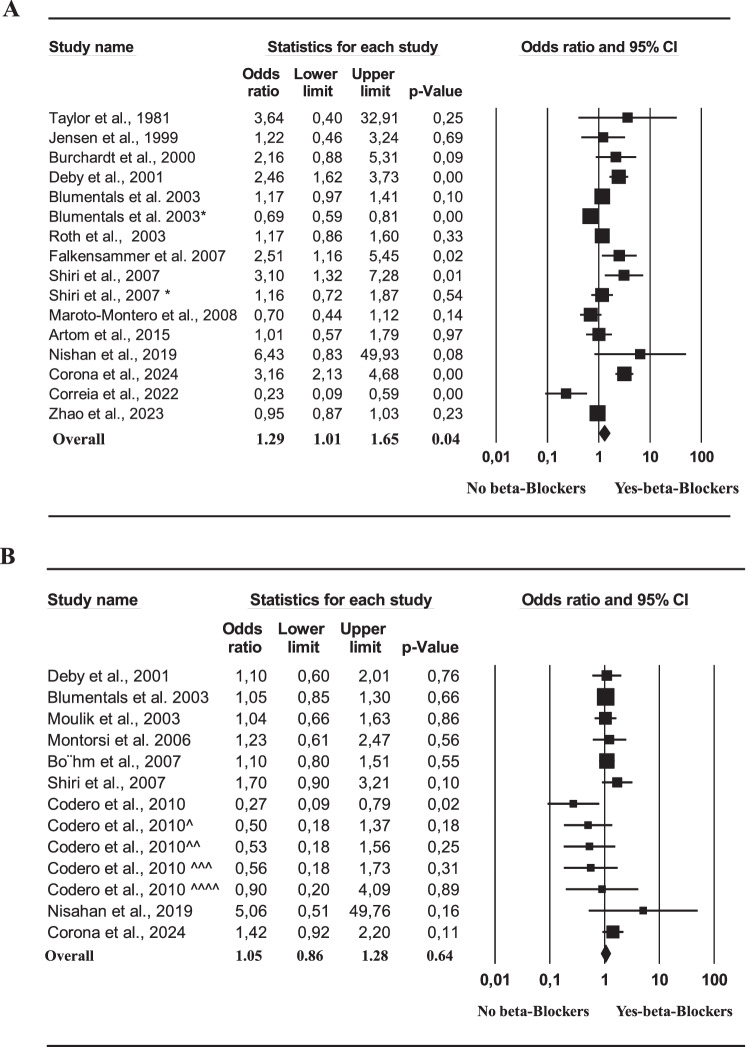
Unadjusted (**A**) and adjusted (**B**) risk of ED in subjects using or not β-blockers as derived from epidemiological studies

**Fig. 2:**
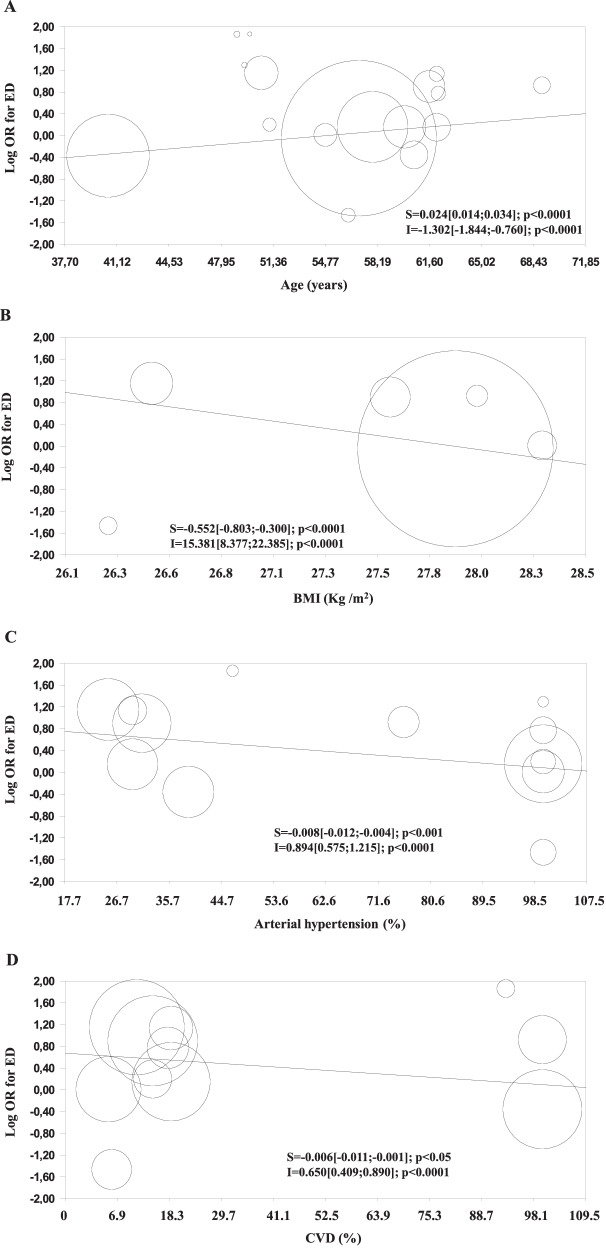
Impact of age (**A**), body mass index (BMI; **B**), arterial hypertension (**C**) and history of cardiovascular diseases (CVD; **D**) on unadjusted risk of ED in patients taking β-blockers

Out of 212, 18 studies were collected on the latter topic [18, 36, 39, 51–65]. In addition, data derived from our population including 3903 patients seeking medical care for ED were also considered (Table 3). The retrieved studies included 264,425 subjects with a mean age of 57.1 years (Table 3). ED was defined according to different methods, and the characteristics of the included studies are reported in Table 3. When unadjusted adjusted data were considered Q and I^2^ were 108.5 and 86.2, respectively (*p* < 0.001). The funnel plot and Begg-adjusted rank correlation test (Kendall’s τ: 0.08; *p* = 0.69) indicated no major publication bias. Overall, the use of BBs was associated with an increased risk of ED (Fig. 1A). The risk of ED-related use of BBs increased with age (Fig. 2A) and decreased according to the increase of other CV risk factors, including enlarged BMI and the prevalence of AH or a past history of CVD (Fig. 2B–D). The latter associations were confirmed even after the adjustment for age (not shown). In line with these observations, when adjusted data were analyzed, no risk of ED was observed when subjects using BBs were compared to controls (Fig. 1B).

**Table 3:** Characteristics of the epidemiological studies included in the meta-analysis

Study (ref.)	# patients	Age (years)	BMI (kg/m^2^)	AH (%)	CVD (%)	ED definition
Taylor et al. [48]	52	49,0	-	65.4	-	Self reported
Jensen et al. [49]	101	51.1	-	100	14.7	Self reported
Burchardt et al. [50]	104	62.2	-	100	18.0	IIEF
Deby et al. [38]	922	61.6	27,6	31.0	14.7	Self reported
Blumentals et al. [51]	3160	57.9	-	-	-	Self reported
Blumentals et al. * [52]	25650	40.6	-	-	-	Self reported
Moulik et al. [53]	763	57.0	-	-	-	Self reported
Roth et al. [54]	353	60.0	-	100	-	IIEF
Montorsi et al. [55]	285	54.4	26.6	49.5	100	IIEF
Böhm et al. [56]	1537	64.9	27.8	73.1	73.6	IIEF
Falkensammer et al. [57]	135	69.0	28.0	76.0	100	IIEF
Shiri et al. [35]	1374	62.1	-	29.4	18.7	Self reported
Maroto-Montero et al. [58]	420	60.6	-	39.0	100	IIEF
Codero et al. [17]	1007	57.9	28.3	100	57	IIEF
Artom et al. [59]	243	54.8	28.3	100	5	Self reported
Nisahan et al. [61]	326	49.0	-	46.6	9.2	Self reported
Correira et al. [61]	385	56.3	26.3	100	5.7	IIEF
Zhao et al. [62]	223805	57.0	27.9	-	-	Self reported
Corona et al. 2024 (unpublished)	3903	50.6	26.5	25.2	11.2	SIEDY

*BMI* body mass index, *AH* arterial hypertension, *CVD* cardiovascular diseases, *ED* erectile dysfunction, *IIEF* international index of erectile function, *SIEDY* structured interview on erectile dysfunction

##### Interventional studies

**Fig. 3:**
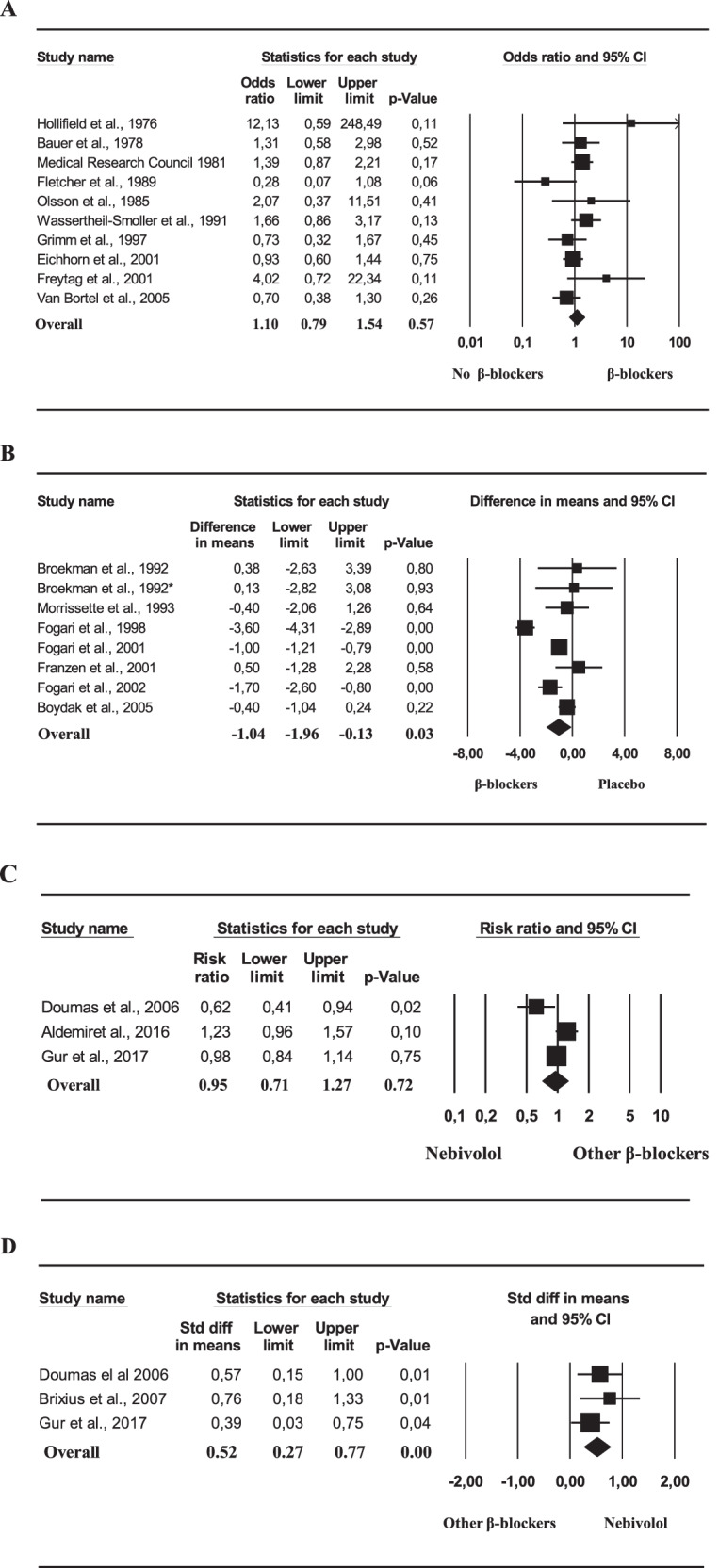
Overall risk of erectile dysfunction (**A**) and weekly frequency of coital intercourses (**B**) related to the use of β-blockers in comparison to controls. Weekly frequency of coital intercourses (**C**) and International Index of erectile function score (**D**) related to the use Nebivolol in comparison to other β-blockers.*newly diagnosed hypertensive patients

Out of 212, 21 RCTs were included in the analysis [7, 20, 21, 37, 66–82]. Among them, 14 were placebo-controlled. Overall, these studies involved 5095 subjects with a mean age of 52.8 years, and mean BMI of 26.2 kg/m^2^, and a mean follow-up of 41.9 weeks (Table 4). When any kind of BBs was compared to placebo or other drugs (i.e., verapamil [68], telmisartan [80] or losartan [81]) no difference in ED rate between groups was detected (Fig. 3A). The same results were confirmed when only placebo-controlled studies were analyzed (OR = 1.21 [0.91;1.59]; *p* = 0.19). However, when the weekly frequency of coital intercourses was investigated, the use of BBs was associated with a reported lower number of sexual intercourses, when compared to placebo (Fig. 3B). To better analyze possible differences in the impact of ED among BB users, we compared the use of nebivolol to others. No difference in ED rate was observed between groups (Fig. 3C). However, when only studies using IIEF were considered, the use of Nebivolol resulted in better outcomes when compared to other BBs (Fig. 3D).

**Table 4:** Characteristics of the interventional studies included in the meta-analysis

Study (ref.)	# patients	Age (years)	Follow up (weeks)	BMI (kg/m^2^)	Type of BBs	Dosage	Comparison	Outcomes
Hollifield et al. [63]	30	46.1	6	—	Propranolol	320 mg/daily	Plabebo	ED rate
Bauer et al. [64]	25	—	104	—	Propranolol	320 mg/daily	Plabebo	ED rate
Fletcher et al. [65]	13	51.2	12	—	Propranolol	80–120 mg/daily	Verapamil	ED rate
Medical Research Council [66]	778	—	104	—	Propranolol	320 mg/daily	Plabebo	ED rate
Olsson et al. [67]	242	59.6	156	—	Metoprolol	100 mg/daily	Placebo	ED rate
Wassertheil-Smoller et al. [68]	470	49.0	24	—	Atenolol	—	Placebo	ED rate
Broekman et al. [69]	26	51.2	6	—	Bisoprolol	5 mg/daily	Placebo	#weekly coital intercourse
Morrissette et al. [70]	16	45.1	4	—	Atenolol	Up to 100 mg/daily	Placebo	#weekly coital intercourse
Fogari et al. [71]	90	46.6	16	—	Atenolol	100 mg /daily	Placebo	#weekly coital intercourse
Fogari et al. [72]	120	56.5	16	24.9	Carvedilol	50 mg/daily	Placebo	#weekly coital intercourse
Franzen et al. [73]	38	—	16	—	Metoprolol	95 mg/daily	Placebo	#weekly coital intercourse
Fogari et al. [74]	55	47.3	16	—	Atenolol	50 mg/daily	Placebo	#weekly coital intercourse
Boydak et al. [75]	43	—	12	24.1	Nebivolol	5 mg/daily	Placebo	#weekly coital intercourse
Grimm et al. [7]	227	55.0	208	—	Acebutol	400 mg/daily	Placebo	ED rate
Eichhorn et al. [76]	2155	60.0	104	28.0	Bucoindolol	Up to 50 mg/daily	Placebo	ED rate
Freytag et al. [77]	298	57.9	24	—	Atenolol	Up to 100 mg/daily	Telmisartan	ED rate
Van Bortel et al. [78]	298	56.0	12	28.1	Nebivolol	5 mg/daily	Losartan	ED rate
Aldemiret al. [79]	23	59.7	12	23.0	Nebivolol	5 mg/daily	Metoprolol	ED rate
Gur et al. [20]	119	55.9	12	27.8	Nebivolol	5 mg/daily	Metoprolol	ED rate/ IIEF
Doumas [36]	44	55.6	26	—	Nebivolol	5 mg/daily	Other BBs	ED rate/ IIEF
Brixius et al. [19]	25	47.8	26	27.7	Nebivolol	5 mg/daily	Metoprolol	IIEF

*BMI* body mass index, *BBs* β-blockers, *ED* erectile dysfunction, *IIEF* international index of erectile function

## Discussion

Poor BP control still represents one of the major issues in the management of people with AH. Data derived from the US have estimated that only 25–35% of subjects with AH are able to reach a stable, good control of BP [83]. Even lower rates have been reported for the European population and minimal control is obtained in low-income countries [84]. Low adherence has been associated with a high risk of BP-related morbidity and mortality, whereas only a 10% increase in the effectiveness of anti-hypertensive treatment due to better drug adherence has been suggested preventing 14,000 deaths a year in the US [85]. Sexual dysfunctions have been considered one of the major problem leading to poor adherence or to withdrawal from anti-hypertensive therapy [86]. Conversely, several studies have documented that adequate management of ED and its successful treatment in hypertensive subjects, can result in improved adherence to BP lowering drugs [87].

International AH guidelines indicate that ACEis, ARBs, CCB, and thiazides should be considered first-line therapy in the management of hypertensive subjects [2, 88–90]. The European Society of Hypertension, along with the European Society of Cardiology (ESC/ESH), also included BBs in the first-line approach [2], whereas this was not reported by other societies [88–90]. Data derived from observational and interventional studies indicate that ACEis, ARBs ABs and CCBs have neutral or even positive effects on erectile function. We here report that these classes of medications might even improve penile blood flow, as demonstrated by an increased flaccid acceleration at PDU. Despite some preliminary negative reports related to the use of thiazides, more recently published data seem not to confirm this negative association. The negative role of centrally acting drugs such as clonidine and α-methyldopa is established but limited controlled trials are available and the current use of these drugs in the management of AH is limited [2, 88–90].

The role of BBs on CVD and ED deserves a better analysis. Long-term prospective studies have clearly demonstrated that BBs reduce mortality of about 20% after myocardial infarction [91]. Similarly a reduction in mortality and morbidity has also been observed in subjects with heart failure [92]. In line with these data, our results showed that BBs play a major role in improving PP and penile vascular flow, as detected by flaccid acceleration in subjects with ED. Accordingly, we previously reported that either higher PP [3] and reduced flaccid acceleration at PCDU [93] represent valid markers of forthcoming MACE in the ED population. As also recognized by as stated by ESC/ESH Guidelines, BBs are not a homogeneous class [2]. In particular, BBs differ in cardioselectivity, sympathomimetic activity, lipid solubility and vasodilating capability [94, 95] (see also Supplementary Table 1). Several intrinsic mechanisms belonging to BBs can explain their potential negative role in erectile function. The main putative negative mechanism of action deals with the possible decrease in penile perfusion pressure due to unopposed α-receptor stimulation. In addition, a possible decrease of testosterone (T) as well as gonadotropins production induced by metoprolol, pindolol, atenolol, and particularly propranolol has also been reported [16]. Indirect effects on T production, due to metabolic negative consequences induced by BBs, can also be advocated. Accordingly, metabolic derangements and obesity are considered one of the main determinants of the so-called *“functional hypogonadism”* [14, 96, 97]. Our data, derived from a large series of subjects with ED, showed that the use of BBs is associated with a lower testis volume as well as decreased T levels without any modifications in LH and FSH concentrations. Finally, a decrease in central sympathetic tone, especially for BBs with higher lipid solubility has also been postulated [87]. In line with this evidence, worse effects have been reported for non-selective BBs, particularly for propranolol [87]. Despite these data, one of the largest meta-analyses published so far showed that although the use of BBs was associated with an increased risk of ED, the absolute risk did not reach statistical significance [98]. In addition, no differences were reported according to BB lipid solubility or when non-selective drugs were compared to later BB generations [98]. In line with these data, a more recently published network meta-analysis, including 19 studies with at least one BB blocker harm, did not find any increased risk of ED [5]. Our data are in line with this evidence. Results derived from epidemiological studies showed that the BB-related risk of ED increased as a function of associated morbidities and age. Conversely, no association was found when adjusted data were investigated. Taken together, these results suggest that BB-associated morbidities, rather than BBs per se, support the link between BBs and ED. Accordingly, when interventional studies were considered, our analysis confirmed the neutral effects of BBs on ED. Interestingly, however, our results indicated a negative relationship between BBs and frequency of intercourse as derived from either the meta-analysis of the interventional trials or from patients seeking medical care for ED at Florence Unit. It should be recognized that knowledge and prejudice about the side effects of BBs can produce anxiety and mood disturbances leading to ED reduced frequency of intercourse and drug withdrawal, the so-called *“nocebo effect”* [19]. Accordingly, our data derived from ED patients, showed that subjects taking BBs more often complained of anxiety and depressive symptoms. Similar considerations can be drawn to explain the relationship between mood disturbances and the reported use of other AH medication classes such as ARBs, CBBs, or diuretics observed in our series.

Nebivolol represents a third-generation BB with cardioselectivity and vasorelaxant activities. However, it has peculiar, specific characteristics since its role in regulating endothelial relaxation is mainly based on the stimulation of nitric-oxide secretion from endothelial cells, rather than on the blockage of α-adrenergic receptors [95, 99]. The interaction of BBs with β_3_ receptors is considered the main pathway related to their possible negative metabolic effects [94]. Interestingly, a recent meta-analysis showed that nebivolol had a lower impact on LDL and HDL cholesterol when compared to other BBs [100]. These specific characteristics can explain, at least partially, our results suggesting a protective role of nebivolol on ED when IIEF data were considered. Similarly, the aforementioned network meta-analysis showed better outcomes on ED when nebivolol was compared to non-vasodilatory BBs [5]. Nevertheless, data related to the effects of nebivolol in comparisons with the other BBs are limited, and the quality of the available studies is rather modest.

Several limitations should be recognized. The quality of the available studies assessing the impact of AH medications on ED is overall modest or poor and only a limited number of well-designed placebo-controlled RCTs are available. In addition, it should be recognized that erectile function data were often not considered the main outcome of the available studies. Finally, in the vast majority of cases, sexual function was derived from patient self reported questions rather than from validated questionnaires.

In conclusion, in line with what was previously reported [5, 13, 30, 38, 87], we showed that BBs represent the class of AH medications more often associated with ED, although better results can be obtained with the use of nebivolol. However, we need to clarify that in many cases, the observed negative effects on ED can be managed with adequate information, preventing negative prejudices and wrong beliefs that can result in worse long-term mortality and morbidity outcomes. In line with what was reported by the ESH [2] and our Society [6], sexual function should be assessed in all patients with AH at diagnosis and after the introduction of specific medications. Although conflicting results have been reported, the use of BBs should be recommended in the presence of specific indications, including angina, post-myocardial infarction, heart failure or when heart-rate control is required [2]. This approach can allow to overcome negative outcomes related to the use of AH drugs and to adequately and timely manage possible side effects.

## Supplementary information

Appendix A

Supplementary Table 1

## Data Availability

The datasets used during the current study are available from the corresponding author on reasonable request.
