# Supplementary material for: Anti-hypertensive medications and erectile dysfunction: focus on β-blockers
Source: Endocrine. 2024 Sep 13;87(1):11–26. doi: 10.1007/s12020-024-04020-x (PMC11739250; doi:10.1007/s12020-024-04020-x)
Supplement: Supplementary file 1 — Appendix A [file 12020_2024_4020_MOESM1_ESM.docx]

**Records removed from the analysis**

Reviews N = 72

No data on sexual function N = 51

Editorial or commentary or letter N =12

Studies conducted in animal models N = 25

**Studies included in quantitative analysis N= 39**

**Records identified on Medline search N= 212**

**Full-text papers assessed for eligibility N= 100**

**Records removed from the analysis**

No data on ED in non beta-blocker treated N = 10

**Interventional studies N= 21**

**Epidemiological Studies N= 18**

**Appendix A.** Flow chart of the included studies
