# Supplementary material for: Anti-hypertensive medications and erectile dysfunction: focus on β-blockers
Source: Endocrine. 2024 Sep 13;87(1):11–26. doi: 10.1007/s12020-024-04020-x (PMC11739250; doi:10.1007/s12020-024-04020-x)
Supplement: Supplementary file 2 — Supplementary Table 1 [file 12020_2024_4020_MOESM2_ESM.docx]

| **Action** | **Type of drug** |
| --- | --- |
| *β_1_ Selective* | *Metoprolol* |
|  | *Atenolol* |
|  | *Acebutol* |
|  | *Nebivolol* |
|  | *Bisoprol* |
|  |  |
| *Non-selective* | *Propranolol* |
|  | *Timolol* |
|  |  |
| *α_1_and β-blocking agents* | *Labetalol* |
|  | *carvedilol* |
|  |  |

**Supplementary Table 1**. Grade of β-receptor selectivity among different β-blockers
